# Paraparesis As a Rare First Presentation Of Primary Hyperparathyroidism-Related Brown Tumor in The Thoracic Spine: Case report and literature review

**DOI:** 10.1016/j.amsu.2021.102197

**Published:** 2021-02-27

**Authors:** Sultan M. Jarrar, Suleiman S. Daoud, Omar F. Jbarah, Iyad S. Albustami

**Affiliations:** Neurosurgery, Department of Clinical Neuroscience, Faculty of Medicine, Jordan University of Science & Technology, PO Box 3030, 22110, Irbid, Jordan

**Keywords:** Brown tumor, Primary hyperparathyroidism, Thoracic spine tumor, Spinal compression, Case report

## Abstract

**Introduction and importance:**

Brown tumor (BT) is defined as osteolytic lesion of an underlying state of hyperparathyroidism. Hyperparathyroidism will activate osteoclasts which initiate active bone resorption foci of lytic-cysts with hemosiderin depositions that pigment it with its characteristic brown pathologic gross appearance. Devastating fractures and injuries can occur to affected bones and surrounding tissue that require emergent intervention and correction.

**Case presentation:**

We present a case of a medically free 31-year-old female patient, who presented complaining of unsteadiness and progressive lower limbs weakness over 40 days of duration. Subsequent lab tests showed elevated PTH levels, along with 3.5 × 1.8 cm heterogeneous soft tissue mass involving the right pedicle on T7 level compressing the corresponding level of the spinal cord. Surgical management aimed to decompress the spinal cord and to obtain a biopsy for histopathologic examination which revealed a brown tumor. Neck ultrasound and Sestamibi scan indicated the presence of hyperactive and hyperplastic parathyroid tissue most suggestive of parathyroid adenoma.

**Clinical discussion:**

Various presentations of Brown Tumor depend on the bone affected, despite the rarity of spinal involvement, yet expanding tumors can manifest either with back pain, radicular pain, paresthesia, weakness, paralysis, or incontinence. The highest incidence rates of spinal brown tumors affect adults over the age of 40. Management goals are to decompress the neuronal tissue emergently and to prevent further bony lytic deterioration.

**Conclusion:**

The objective of this study is to provide an overview of primary hyperparathyroidism-related spinal brown tumors, presentation, and summary of previously reported similar cases in the literature.

## Highlights

• Brown tumors are rare manifestation of today's primary hyperparathyroidism clinical presentation and context when compared with secondary hyperparathyroidism.
• Up to 3% of patients with primary hyperparathyroidism develops BTs, the incidence is furtherly declining due to screening and health assessment programs.
• Brown tumors are destructive lesions that could be single or multiple at any site involving long bones, hands, mandible, pelvis and ribs, which are way more common sites than the spine.
• Almost half of Primary Hyperparathyroidism-Related Brown Tumors of the spine were found to affect the thoracic region. While sacral region was least to be affected.

## 1 Introduction

Brown tumor is an osteopathic reactive lesion that results from persistent elevation of parathyroid hormone, which aids calcium and phosphate homeostasis in the body. Hyperparathyroidism is either a primary parathyroid pathology or could be a compensatory mechanism in chronic calcium wasting conditions like chronic kidney diseases; both primary and secondary hyperparathyroidism were found responsible for the abnormal progressive bone remodeling activities seen in brown tumors [1,2].

The patient had a brown tumor involving the seventh thoracic vertebra T7 with bilateral lower limbs weakness and numbness as a rare first presentation for primary normo-calcemic hyperparathyroidism. We have reported the case presentation, relative physical examination, management, and a literature review covering the 30 reported cases in the literature. This case report was reported in line with SCARE 2020 criteria [3].

### 1.1 Case presentation

We reported a case of a 31-year-old female who is medically free, presented with a history of gait unsteadiness and bilateral lower limbs weakness and numbness of 40 days duration. She addressed difficulties in standing and sitting when she is lying down. Her symptoms were progressive in a manner that affected her productivity and daily activities.

The patient denied having difficulties in passing urine or stool. No reported history of fever, weight change, or trauma, and is not on any regular medications or supplements. The patient had no history of personal nor familial history of endocrine tumors. She used to take over-counter supplemental vitamins.

Clinically she was slim presented in a wheelchair with difficulty rising up to a standing position. Upon physical exam, the patient's vital signs were within normal ranges. She was awake and oriented. Cranial nerves were intact. Examination of her limbs showed full strength in upper limbs and −4/5 strength on her lower limbs. Deep tendon reflexes were 3+ on knee jerk and ankle jerk. The lower limbs were hypertonic. Babinski test showed upward going toes. Fine and pinprick sensations were intact. Digital rectal examination had normal sensation, normal tone, but weak power. No skin lesions were noted.

Her blood tests showed sky-high parathyroid hormone levels of 862 pg/ml (normal range is 15–65 pg/ml). Despite the elevated parathyroid hormone levels, Calcium, phosphorus, vitamin D, thyroid hormone levels and kidney function results were within the normal ranges. Also, a complete paraneoplastic markers assessment turned negative. Subsequently, she underwent parathyroid ultrasound which revealed evidence of well-defined hyperechoic mass lesion in the left anterior neck region, measuring about 2*0.5*0.6 cm. This lesion was highly suspicious for parathyroid adenoma.

**Fig. 1:**
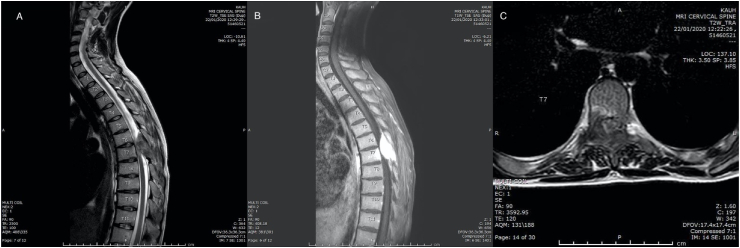
(A)(B) sagittal cervicothoracic MRI T2/T1 sequences showing large osteolytic bone lesion 3.5 × 1.8 cm, at level of T7. Significantly distorted the posterior elements of the corresponding vertebral spine. (C) axial MRI cut scan at level of T7 showing expansile osteolytic mass, involving the right pedicle and posterior arch, compressing the spinal canal and dislocating its content to the left.

Whole spine MRI was performed and revealed heterogenous soft tissue mass lesion involving the right pedicle, lamina, transverse process, and inferior articular process of T7, as well as the right posterolateral aspect of T7 vertebral body. This lesion appeared predominantly isointense on T1/T2WI with vivid enhancement on postcontrast images measuring about 3.5 × 1.8 cm. This lesion was shown to compress and displace the spinal cord from the right which causing compressive myelopathy on the corresponding level of the spinal cord (Fig. 1).

**Fig. 2:**
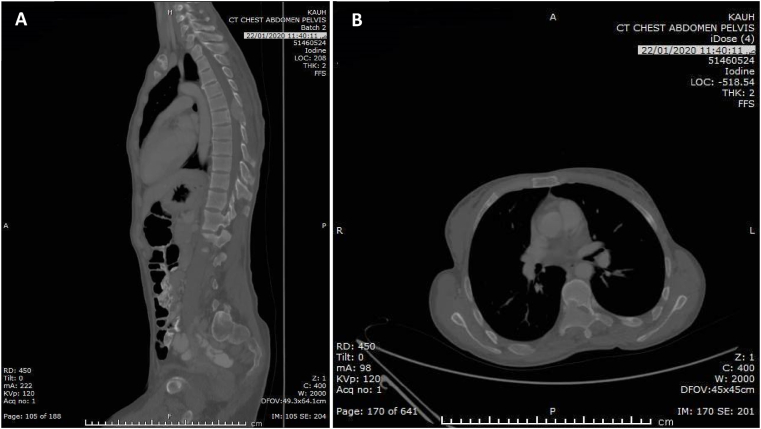
(A) sagittal chest abdomen pelvis CT showing expansile bony mass involving the body, right pedicles and posterior arch of T7. (B) axial T7 CT showing epidural soft tissue component extending from T6-T7 compressing the spinal canal, dislocating its content to the left and scalloping the T7 vertebral body.

Contrasted multiple axis Computed Tomography of neck, chest, abdomen, and pelvis showed well defined enhancing mass lesion in the left anterior neck region. In addition to an expansile bony mass involving the body, right pedicles and posterior arch of T7 was noted. The lesion was associated with epidural soft tissue component extending from T6-T7 compressing the spinal cord and scalloping the T7 vertebral body(Fig. 2). Of note, there were generalized osteogenic changes involving the ribs, clavicle, scapula, and the examined spine.

Bone scan showed several foci of increased and decreased uptake in the axial bone skeleton mainly in the ribs and spine consistent with mixed osteolytic/osteoblastic bony lesions suggestive of brown tumors.

The patient expressed her distress of the emergent weakness that significantly impaired her ability to maintain daily activities, therefore surgery aimed to decompress and to stabilize the thoracic spine to allow for motor function improvement, as well as to obtain histopathological examination.

**Fig. 3:**
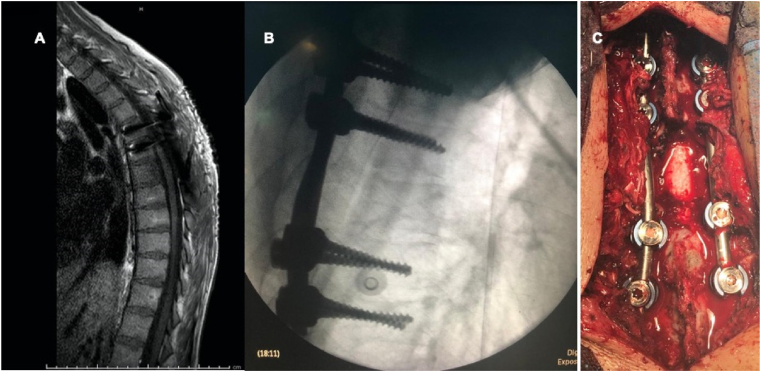
(A) sagittal MRI demonstrates segmental spinal instrumentation with T5-T9 transpedicular screws fusion. (B) lateral plain XRAY demonstrate T5-T9 transpedicular screws fusion. (C) intra-operative view of T5-T9 transpedicular screws fusion.

The patient was prepared for surgery having her consent. We performed A posterior midline approach that involved T7 decompressive laminectomy, and removal of the tumor. For stabilization, we performed T5-T9 transpedicular screws fusion (Fig. 3). The surgery was conducted by team of consultant neurosurgeon and senior neurosurgery resident at tertiary university hospital. Post operatively, the patient had no complications and her lower limbs regained full strength and hypertonia was significantly improved.

Histopathological exam showed portions of bone containing a cellular spindle cell proliferation with abundant brown, granular and globular material and scattered groups of multinucleate giant cells of the osteoclast type, no malignant degeneration was noted. In the context of high parathyroid hormone levels, a diagnosis of brown tumor of hyperparathyroidism was made.

Work up for parathyroid adenoma continued with a Sestamibi scan that showed no scintigraphic evidence of parathyroid adenoma, however, findings were compatible with false negative test. The patient was referred for general surgery team for further management of her parathyroid lesion.

## 2 Discussion

Extracellular levels of calcium depend on regulatory mechanisms and signaling hormones. Parathyroid hormone plays an indispensable adjusting role in calcium homeostasis, tightly controlling calcium fluxing between bones, kidneys, intestine, and plasma [1].

Abnormally sustained elevation of parathyroid hormone levels is considered a state of hyperparathyroidism, classified into primary, secondary, and tertiary; the latter two subtypes are sequalae of long-standing medical conditions especially chronic kidney diseases. Primary hyperparathyroidism manifest as either the classical variant of hypercalcemia or as normo-calcemic variant, the former variant is mostly attributed to parathyroid benign adenomas, malignant adenomas or parathyroid hyperplasia, while the normo-calcemic variant is of idiopathic origin, usually remains subclinical but has a potential to progress into hypercalcemic state and to cause bone targeted damage [2].

Biologically, parathyroid hormone stimulates bone resorbing activity of osteoclasts,thus hyperparathyroidism clinically manifest as metabolic bone disease; leads to abnormal structural changes and bone remodeling; includes osteopenia, salt & pepper skull, subchondral resorption, subperiosteal bone resorption-acroosteolysis, and focal lytic lesions [4].

Brown tumors (BT), also known as osteitis fibrosa cystica are well demarcated, benign, rapidly growing lesions of lytic-cysts due to hyperparathyroidism, yet BTs are rare manifestation of today's PTPH clinical presentation and context when compared with secondary hyperparathyroidism. Researchers estimate that up to 3% of patients with PHPT develops BTs, the incidence is furtherly declining due to screening and health assessment programs [5].

Osteoclast-like multi-nucleated giant cells and hemosiderin laden macrophages cells are responsible for the radiological and gross features of brown tumor. The structural texture of brown tumor combine a hemorrhagic, cystic and solid components that constitute the distinctive diagnostic features of BTs on imaging modalities [6,7]. Brown tumor's x-rays demonstrate a multifocal lucent lesion with cortical thinning and soap-bubbly appearance [5]. These bone destructive lesions could be single or multiple at any site involving long bones, hands, mandible, pelvis and ribs, which are way more common sites than the spine [8]. Table 1 shows a list of 30 case reports including this one, that reported a spinal involvement with a PHPT-related BT.

**Table 1:** Previously reported cases of primary hyperparathyroidism-related brown tumors.

#	Author	year	Sex	Age	Level	Clinical	Management
1	Shaw and Davies [14]	1968	F	58	T10	Paraparesis, urinary retention	surgical decompression, parathyroidectomy
2	Shuangshoti et al. [15]	1972	M	32	L4	Paraparesis, radicular pain	surgical decompression,parathyroidectomy
3	Siu et al. [16]	1977	F	64	T10	Paraparesis, urinary retention	surgical decompression, parathyroidectomy
4	Sundaram and Scholz [17]	1977	F	63	T10	paraplegia and urinary retention	surgical decompression, parathyroidectomy
5	Ganesh et al. [18]	1981	M	40	T2	Paraparesis, radicular pain	Parathyroid adenoma excision only
6	Yokota et al. [19]	1989	F	58	T5	Paraparesis, numbness	surgical decompression, parathyroidectomy
7	Daras et al. [20]	1990	F	54	T9	Paraparesis	surgical decompression
8	Kashkari et al. [21]	1990	F	51	T6-T7	Paraparesis	surgical decompression Parathyroidectomy
9	Sarda et al. [22]	1993	F	23	T3-T4	Paraplegia, radicular pain	surgical decompression, parathyroidectomy
10	Motateanu et al. [23]	1994	M	57	L4-5	Radiculopathy	surgical decompression, parathyroidectomy
11	Ashebu et al. [24]	2002	F	27	C6	Bilateral renal calculi, lethargy, weakness, multiple brown tumors	Parathyroidectomy + Orthosis
12	Mustonen et al. [25]	2004	M	28	L2	Radiculopathy, numbness	Resection of parathyroid adenoma only
13	Haddad et al. [26]	2007	F	62	T2-T3	Paraparesis, numbness	surgical decompression, parathyroidectomy
14	Khalil et al. [27]	2007	M	69	L2	Radiculopathy	surgical decompression
15	Altan et al. [28]	2007	F	44	S2	Radiculopathy, low back pain	surgical decompression parathyroidectomy
16	Hoshi et al. [29]	2008	F	23	Sacrum	ureterolithiasis, Radiculopathy	Resection of parathyroid adenoma only
17	Kerstens et al. [30]	2013	M	55	C7/L3	Weight loss, bone pain	Parathyroidectomy
18	Lee et al. [12]	2013	M	65	L1-L2	Low back pain, radicular pain	surgical decompression parathyroidectomy
19	Khalatbari and Moharamzad [6]	2014	F	52	C6	Radiculopathy, neck pain	Parathyroidectomy, surgical decompression
20	Khalatbari and Moharamzad [6]	2014	M	38	T7	Paraparesis, sphincter dysfunction	Parathyroidectomy, surgical decompression
21	Khalatbari and Moharamzad [6]	2014	M	16	L2	Paraparesis, sphincter dysfunction	Parathyroidectomy, surgical decompression
22	Khalatbari and Moharamzad [6]	2014	F	46	L3	Paraparesis, low back pain	Parathyroidectomy, surgical decompression
23	Sonmez et al. [8]	2015	M	50	T9	Paraparesis, sphincter dysfunction	surgical decompression, Parathyroidectomy
24	Alfawareh et al. [13]	2015	F	26	C2	axial neck pain	Parathyroidectomy + Orthosis
25	Heidarpour et al. [11]	2017	M	33	T4	paraplegia, GI bleeding	parathyroidectomy, surgical decompression
26	Carta et al. [9]	2019	M	48	C2	neck pain and quadriparesis	non-operative orthosis with parathyroidectomy
27	Hu et al. [10]	2019	F	50	T9	Paraparesis, thoracic back pain	parathyroidectomy
28	Hammou et al. [31]	2020	F	65	C5	Quadraparesis	surgical decompression, Parathyroidectomy
29	Shaaban et al. [32]	2020	M	37	T4-T5	Paraparesis	Emergent decompression and parathyroidectomy
30	this one	2021	F	30	T7	Paraparesis	Surgical decompression and parathyroidectomy

31 spinal lesions identified as PHPT-related brown tumors were reported in 30 case-reports, among these, 15 lesions (48.38%) were found to affect the thoracic region which is the most common site for PHPT-BTs. The lumbar region is the second most affected site with 8 (25.80%) reported lesions. While the cervical and sacral regions were the least to be affected, 6 (19.35%), 2 (6.45%) respectively.

Brown tumor complications exceed the sole risk of pathological fractures following cortical thinning [7,9,10], since spinal BTs involving the vertebral body and posterior vertebral elements carry a potential risk of extension into the spinal canal or compressing its contents; that render patients with neurological impairments and deficits. Emergent presentations of spinal BTs require emergent decompressive interventions to relieve the nerve tissue; among these emergent presentations, asymptomatic PHPT patients show to have advanced bone damage that correlates with their sky-high levels of PTH [[11], [12], [13]].

## 3 Conclusions

Although of the benign nature of brown tumor, it can cause devastating fractures due to cortical thinning. In cases of spinal vertebrae involvement, brown tumors can cause neurologic symptoms of paresthesia, paresis, or paralysis because of spinal compression. In symptomatic cases it is recommended to ensure tumor resection, spinal decompression and stabilization, along with medical or surgical parathyroid intervention to control the underlying cause.

## Methodology

This case-report has been reported in line with the SCARE 2020 criteria.

## Source of funding

This study was not funded

## Consent

Written informed consent was obtained from patient for publication of the case report and any related images. A copy of the written consent is available for review by the editor-in-chief of this journal on request.

## Author contribution

This work was carried out in collaboration between all authors. Authors SMJ and SSD designed the study. Author ISD managed the literature searches, data collection and wrote the first draft of the manuscript. Author OFJ managed the literature searches and completed the final draft. All authors read and approved the final manuscript.

## Ethical approval

It is not applicable.

## Sources of funding

The authors declare that this case report and literature review are not funded.

## Author contribution

This work was carried out in collaboration between all authors. Authors SMJ and SSD designed the study. Author ISD managed the literature searches, data collection and wrote the first draft of the manuscript. Author OFJ managed the literature searches and completed the final draft. All authors read and approved the final manuscript.

## Registration of research studies

Name of the registry: N/a.

Unique Identifying number or registration ID: N/a.

Hyperlink to your specific registration (must be publicly accessible and will be checked): N/a.

## Guarantor

Sultan M. Jarrar, MD, Assistant professor of Neurosurgery, Department of clinical neuroscience.

Faculty of medicine, Jordan University of Science & Technology PO Box 3030 zip code 22110. IrbidJordan, Telephone: 00962 790033567, Email: smjarrar@just.edu.jo.

## Provenance and peer review

Not commissioned, externally peer-reviewed.

## Declaration of competing interest

The authors report no conflict of interest concerning the materials or methods used in this study or the findings specified in this paper.
